# Practical Papers on Diseases of the Throat and Air Passages

**Published:** 1866-10

**Authors:** Edward B. Stevens

**Affiliations:** Professor of Materia Medica in the Miami Medical College of Cincinnati


### Practical Papers on Diseases of the Throat and Air Passages.

BY EDWARD B. STEVENS, M. D.,
Professor of Materia Medica in the Miami Medical College of Cincinnati.

*Applications of the Laryngoscope.*—In the July number of this journal we attempted to explain the general principle of the *laryngoscope*, and the manner of its mechanical application. Accompanying wood-cuts sufficiently illustrated the whole *modus operandi*, together with outlines of the appearance of the laryngeal opening as seen in health. Without any endeavor to be elaborate, we wish to indicate to our readers who are not as yet familiar with this improvement in laryngeal diagnosis, how it may become an important help. And in the first place we may say that it is so, both in a positive and negative manner; for while the examination by the laryngoscope may reveal well defined local diseases sufficient to account for existing symptoms, and therefore indicate precisely the plan of treatment needed for the individual case, it may also reveal the fact just as positively that no local disease whatever is present, and hence of course forbidding the endless round of swabs, probangs and caustics which have often found their way heretofore into innocent throats in the empirical hope of effecting a cure of something not well understood, but only vaguely surmised.

Modifications of the voice, and cough, are the immediate effects of morbid growths and other lesions of the laryngeal structure.

Minute fibroid bodies, or minute polypi, find their attachment on the vocal cords, or upon the parts so immediately neighboring as to interfere with their normal action—and hence as a general result there is more or less complete loss of voice, and this coming on so gradually that the patient and physician are misled into the belief that it is the legitimate result of catarrhal trouble, not involving change of structure. Sometimes instead of the aphonia, or with it, there is an irritable cough, provoked by the presence of what is simply and truly a foreign body.

In the June number of this journal, Dr. Bruhl, of this city, gives the substance of one of these interesting cases. It is taken from *Berliner Woch. Klinik*, and was operated upon by Dr. Gottstein, of Breslau. In this case there was first dry cough and hoarseness, with complete aphonia after two years, insufferable respiratory difficulty and dyspnœa. Dr. Gottstein found on the laryngoscopic examination a number of small granular bodies or papillomata attached to the posterior wall of the epiglottis, on the ventricular bands and extending downwards to the vocal cords and more or less blocking up the laryngeal cavity. These bodies were removed by proper instrumental interference, and in a very short time the patient was completely restored to her voice and general health. (See *Lancet and Observer*, page 344 *et seq.* of this year.) This case is a very instructive one, and is typical of many others to a greater extent than is generally supposed.

In his Prize Essay for the American Medical Association for 1865, Dr. Ellsberg of New York, has given a large number of cases in illustration of the presence and effects of morbid growths in the air passages. One of these is particularly interesting, especially in view of the remarkable quantity constituting the mass of morbid tissue; and occupying so much of the laryngeal cavity as to hang below the vocal cords and down into the trachea. In this case there was a harrassing hacking cough, and the voice was only a labored whisper. Dr. Ellsberg was compelled, in this case, to *educate* the fauces and epiglottis to laryngoscopic manipulations for a length of time, before operative proceedings could be resorted to. He finally, and by repeated piecemeal, removed a growth which he estimates to be equal to a hen's egg in bulk. The operation was a complete success, but vocalization was only partially restored.

A large number of additional cases are reported in this essay; most of them, perhaps, more practically useful, if not of so striking a character. Quite a number of them are cases in which there is more or less complete aphonia, and a degree of cough. Several of them are produced by the presence of small polypi having their attachment to the vocal cords, or by small fibroid growths, acting essentially in the same manner, so far as they prove laryngeal obstructions; and for the most part the removal of these growths, whatever their character, was uniformly attended with a relief of the attendant symptoms.

This is not properly the place to detail the mode of extirpating morbid growths of this character. Laryngoscopists, however, have devised exceedingly ingenious instruments adapted for these delicate operations—in the form of forceps, probes and scissors. Some of them, indeed, may be conveniently removed with chromic acid, or other escharotics; the apparatus being necessarily delicate, but the principle of surgical therapeutics being precisely as for like procedures in other localities.

But after all, in laryngoscopy, the great advantage of our new means of diagnosis is not particularly to demonstrate the presence of these local conditions of disease or growths; but it is a wonderful advance in our art that we are herehy enabled to arrive at precision in our knowledge of any individual case. Heretofore all pathological conditions of the larynx have been only arrived at by virtue of the rational signs. Sometimes these have indicated a correct plan of therapeutics—quite as frequently our proceeding has been absolutely empirical, and when a cure has been effected we could scarcely be sure whether it occurred from the remedy, or from natural causes. Now we have a case of laryngeal trouble—cough, dyspnœa, aphonia—presented to us, and we proceed to explore the physical condition of the structure with the same expectation of certainty as any other organ, the condition of which we can arrive at a positive knowledge. If we find the distinct results or presence of inflnmmation, we may use our swab, or inhalation with a comfortable sense of certain propriety. If we discover a polypus or other mischief making growth, we may apply a direct escharotic or resort to proper surgical appliance, aided by the laryngeal mirror; or if finally we discover *no* local disease we may at once relieve our patient from all annoyance of probangs, gargles or caustic, and address ourselves to the proper indications of constitutional treatment.

Laryngoscopy being mainly in its infancy, it is probable that its cultivators may be tempted to an exaggerated view of its importance in some respects. It is, however, more probable that with its maturity as a branch of diagnostic exploration, we shall by and by come to find new applications, and an increased range of pathological indications.—*Cincinnati Lancet and Observer.*

